# The Effects of Radioimmunotherapy and Antibiotics on Biofilm‐Associated Implant Infections in a Preclinical Rat Model

**DOI:** 10.1002/jor.70216

**Published:** 2026-05-07

**Authors:** F. Ruben H.A. Nurmohamed, Kevin J. H. Allen, Mackenzie E. Malo, Connor Frank, Colleen Nesbitt, J. Fred F. Hooning van Duyvenbode, Michelle Buijs, Berend van der Wildt, Alex J. Poot, Marnix G. E. H. Lam, Jos A. G. van Strijp, Johannes M. van der Merwe, H. Charles Vogely, Harrie Weinans, Bart C. H. van der Wal, Ekaterina Dadachova

**Affiliations:** ^1^ College of Pharmacy and Nutrition University of Saskatchewan Saskatoon Canada; ^2^ Department of Orthopedics University Medical Center Utrecht Utrecht The Netherlands; ^3^ Department of Orthopedics University of Saskatchewan Saskatoon Canada; ^4^ Department of Radiology and Nuclear Medicine University Medical Center Utrecht Utrecht The Netherlands; ^5^ Department of Medical Microbiology University Medical Center Utrecht Utrecht The Netherlands; ^6^ Department of Biomechanical Engineering Delft University of Technology Delft The Netherlands; ^7^ Department of Orthopedic Surgery Leiden University Medical Center Leiden The Netherlands

**Keywords:** 177Lutetium, prosthetic joint infections, radioimmunotherapy, rat pre‐clinical model, S. aureus, vancomycin

## Abstract

Indwelling medical implants are susceptible to developing biofilm‐associated infections that are notoriously difficult to eradicate. These persistent infections often cannot be resolved with antibiotics alone and typically require surgical intervention for effective management. An alternative approach is radioimmunotherapy (RIT) which uses specific antibodies linked to radioisotopes to selectively destroy bacteria. This antimicrobial approach bypasses traditional antibiotic mechanisms, and RIT is hypothesized to enhance outcomes beyond antibiotic therapy alone. RIT bactericidal effects were studied in Wistar Han rats fitted with femoral rod implants covered by matured 3‐day biofilms. The rats (six per group) were treated with either: RIT with ^177^Lu‐labeled 4497 antibody to *S. aureus* teichoic wall acid (WTA) (116.6 MBq/kg), or vancomycin (88 mg/kg), or combination of RIT (116.8 MBq/kg) and vancomycin, or left untreated. To evaluate efficacy, bacterial counts were taken from the joint capsule, bone, and implant after 7 days. Uptake and biodistribution were assessed via non‐invasive *in vivo* SPECT/CT imaging and *ex vivo* gamma counting. Single administration of RIT achieved a 2.7‐log (99.78%) reduction of bacterial burden in the infected joint capsule, had no effect on the infected femur, and resulted in 72.5% reduction of bacterial burden on the infected implant when compared to untreated controls. RIT reduced bacterial burden and inflammation in experimental PJI with no side effects. These findings underscore the potential of RIT in the treatment of infected indwelling devices and warrant further study.

## 1 Introduction

Biofilm‐associated implant infections pose clinical challenge due to chronic character, increased antibiotic resistance and host evasion mechanisms [1, 2]. A classic example of a biofilm‐associated implant infection is periprosthetic joint infection (PJI), which occurs following arthroplasty and represents a serious complication due to its persistent and difficult‐to‐treat nature. Biofilms are structured aggregations of bacteria including a polymer matrix composed of exopolysaccharides, lipids, proteins, DNA, and wall teichoic acids (WTA) [3]. One of the most prevalent pathogens in PJI is *Staphylococcus aureus* with a 30%–40% incidence. This pathogen is also notorious for its biofilm formation [4, 5]. Treatment of biofilm‐associated implant infections is accomplished through surgery and a systemic antibiotics [6]. However, complete recovery remains challenging and, given the growing concerns for antimicrobial resistance, alternative treatment strategies should be explored.

**Figure 1:**
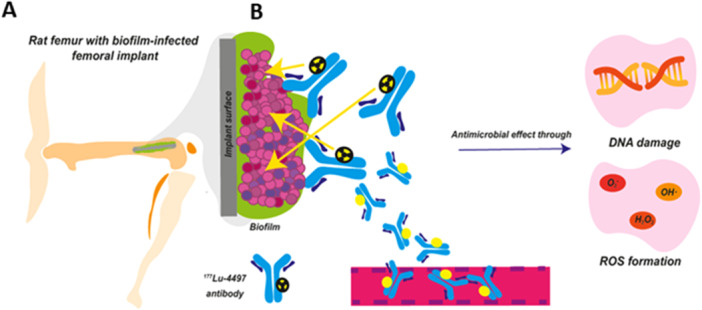
Simplified schematic illustration of the in vivo biofilm‐associated infection model and the mechanism of action of radioimmunotherapy (RIT). (A) In this preclinical model, a mature *Staphylococcus aureus* biofilm was maturated over 3 days; (B) The RIT consists of an anti‐β‐GlcNAc wall teichoic acid (WTA) IgG1 antibody radiolabeled with Lutetium‐177, a β‐emitter. This radioimmunoconjugate specifically targets both gram‐positive bacteria (pink circular bodies) and their biofilm matrix. Following systemic circulation, the radiolabeled antibodies will bind specifically to the bacteria and biofilm, exerting antimicrobial effects through induction of single‐ and double‐strand DNA breaks as well as generation of radiation‐induced reactive oxygen species (ROS).

Targeted radiation using radiolabeled antibodies, known as radioimmunotherapy (RIT), offers a possible alternative to antimicrobial therapy by employing ionizing radiation to eradicate bacteria ^7^. In RIT, therapeutic radionuclides are attached to antibodies with high affinity for bacteria‐specific antigens. Ultimately, this localized ionizing radiation induces DNA damage, which triggers death of bacteria (Figure 1). In addition, reactive‐oxygen‐species (ROS) will be generated, leading to indirect oxidative damage to critical bacterial components [7, 8].

In our previous work, we demonstrated high specificity of 4497‐IgG1 antibody (anti‐β‐GlcNAc WTA) in a rat model with a biofilm‐associated implant infection [9]. It is important to note that while the application of RIT is novel in the PJI field, it is an established treatment modality for certain malignancies [10, 11, 12]. Following our encouraging initial findings, we hypothesized that a single RIT administration might have a comparable effect on bacteria biofilm in a rat model as a standard of care course of antibiotics. In this study, a β‐emitting radionuclide Lutetium‐177 [^177^Lu] with 6.7 days physical half‐life was selected for labelling a WTA‐targeting 4497 antibody because of its well‐established clinical use and broad availability, facilitating potential translational implementation.

## 2 Methods

### 2.1 Antibody, Radionuclides and Radiolabeling

The 4497‐antibody (Mut H + Y HuIgG1‐anti‐WTA‐4497) was produced and purified as in [13]. It was conjugated to the bifunctional chelator S‐2‐(4‐Isothiocyanatobenzyl)−1,4,7,10‐tetraazacyclododecane tetraacetic acid (p‐SCN‐Bn‐DOTA, Macrocyclics^TM^, Plano, TX, USA) as in [14, 15] and radiolabeled with ^177^Lu (McMaster University, Canada) as previously described [16].

### 2.2 Implant Infection Induction and Biofilm‐Associated Implanted Infection Rat Model

*S. aureus* was used to grow biofilm on the femoral rod implants [17], USA300 LAC (AH4802) strain [18]. The infection of titanium alloy implants (medical grade 23, ELI, Ti6AI4V) was induced as in [19]. The *in vivo* experiments were approved by the Animal Research Ethics Board of the University of Saskatchewan, Canada (protocol AUP20230036) and conducted according to the ARRIVE guidelines. A biofilm‐associated implant infection was established using a single femoral implant in each of 24 Wistar Han male rats (Charles River), aged approximately 13–14 weeks as described in [19].

### 2.3 RIT of Implant‐Associated Infection in a Rat Model

The rats were randomly assigned to 4 treatment groups consisting of 6 male rats each. The colony forming units (CFU) counts and single photon emission computed tomography/computed tomography (SPECT/CT) imaging were performed by personnel blinded to treatment groups to avoid interpretation bias. The treatment groups included a NaCl 0.9% IV injection (control); RIT (single intravenous injection of 116.6 MBq/kg ^177^Lu‐4497, averaged for *n* = 6), vancomycin (88 mg/kg of vancomycin hydrochloride (Fresenius Krabi) administered intraperitoneally, q12h, until termination); and a combination therapy consisting of a single IV dose of RIT (116.8 MBq/kg ^177^Lu‐4497, averaged for *n* = 6) with vancomycin therapy (88 mg/kg, IP, q12h, until termination). The choice of RIT dose was based on our previous study in the same infection model where the average dose of 112 MBq/kg resulted in minimal systemic and hematologic toxicity [19]. The 4% difference in this study between the intended and actual doses is caused by a different model of syringes for injection available for the current study.

### 2.4 SPECT/CT Imaging

^177^Lu‐4497 distribution during the 7‐day treatment period was evaluated with SPECT/CT imaging at 24, 96, and 168 h post‐RIT administration on the VECTor^4^CT scanner (MILabs) as in [9, 19].

### 2.5 Evaluation of RIT Antimicrobial Efficacy

At 7 days post‐RIT administration the rats were sacrificed, and the joint capsule and the femur with biofilm‐infected implant were aseptically collected, and CFUs were obtained as in [19]. During calculations of mean CFUs and standard deviations for any treatment group, the NDC (no colonies detected) were treated as a 0 value for the calculation of means and standard deviations.

### 2.6 ^177^Lu‐4497 Biodistribution and Short‐Term Toxicity

After the 7‐day treatment period, the rats were humanely sacrificed, and biodistribution was conducted as in [19]. The percentage of the injected dose per gram (%ID/g) in organs and implant was calculated:

$$\%\;\mathrm{Injected}\;\mathrm{dose}\;\mathrm{per}\;\mathrm{gram}\;\left(\%\frac{\mathrm{ID}}{\mathrm{gram}}\right)=\frac{\text{Counts per minute}\;\left(\text{CPM}\right)}{\text{Organ weight}\;\left(\text{gram}\right)\times \left(20\;\times \;\text{CPM standard}\right)}\times 100\%$$

Blood was collected via cardiac puncture at sacrifice, and plasma was analyzed to assess short‐term systemic toxicity using a liver panel (aspartate aminotransferase (AST), alanine transaminase (ALT), and albumin) and a renal panel (creatinine and urea). The results were compared against clinical reference values provided by Charles River Laboratories [20].

### 2.7 Statistical Analysis

Sample size calculation was based on bacterial assessment of the implant performed in [19], and it was determined that a minimum of six animals per group was required. The effect size (*d* = 2.099) was calculated comparing the RIT group (116.6 MBq/kg) to the control group, using a statistical power of 95% and an alpha level of 0.05 (G*Power version 3.1.9.7). Means and standard deviations were plotted using GraphPad Prism 7.4. A two‐way ANOVA was performed to evaluate differences in antimicrobial efficacy and *ex vivo* biodistribution. Normality was checked by calculating the standardized residuals and using a QQ plot for interpretation. Dunnett's test which compares all groups to a reference group (untreated controls in our study) was used for post hoc analysis.

## 3 Results

### 3.1 Single RIT Administration Demonstrated Comparable to Vancomycin Effects on Planktonic Cells in Capsule and Biofilms

The mean implant infection burden at the start of the experiment was 3.1 × 10⁷ CFUs. Table 1 and Table S1A–D display the CFUs in joint capsule, femur and on implant in all groups at the time of experiment termination 7 days post RIT. The single injection of ^177^Lu‐4497 reduced mean bacterial count in joint capsule by 2.7 log, or 99.78% (*p* = 0.02) reduction compared to control group. In contrast, the surrounding femoral bone exhibited no reduction compared to control (*p* = 0.10). The femoral implant exhibited 0.4 log, or 72.5% (*p* = 0.08) reduction compared to control. In the comparator vancomycin group no detectable colonies were observed in the joint capsule (*p* < 0.0001). The effect on the biofilm‐covered femoral bone and femoral implant was much less pronounced with 2.2 log, or 99.41% (*p* = 0.04) and 0.8 log, or 85.54% (*p* = 0.06) bacterial reduction compared to control, respectively. Similar to vancomycin group, the RIT plus vancomycin group did not have any detectable colonies in the joint capsule (*p* < 0.0001). The femoral bone and femoral implant exhibited 0.5 log, or 67.93% (*p* = 0.10) and 2.0 log, or 99.09% (*p* = 0.04) bacterial reduction compared to control, respectively. Taken together, these results demonstrate that a single RIT administration was comparable to a 7 day vancomycin course effects on planktonic cells in capsule and biofilms on the implant though neither treatment was very effective against biofilms.

**Table 1:** Mean CFUs in treatment groups.

Treatment group	Joint capsule, CFU	Femur, CFU	Implant, CFU
Control	(4.3 ± 4.4) x 10^7^	(4.0 ± 2.3) x 10^6^	(4.0 ± 7.6) x 10^6^
RIT	9.3 × 10^4^ ± 2.3 × 10^5^ (*p* = 0.02)	(2.0 ± 1.9) x 10^7^ (*p* = 0.10)	(1.1 ± 2.3) x 10^6^ (*p* = 0.08)
Vancomycin	0 (*p* < 0.0001)	(1.7 ± 4.1) x 10^5^ (*p* = 0.04)	(4.1 ± 9.2) x 10^5^ (*p* = 0.06)
RIT+Vancomycin	0 (*p* < 0.0001)	(1.3 ± 2.5) x 10^6^ (*p* = 0.10)	(3.6 ± 1.7) x 10^4^ (*p* = 0.04)

*Notes:* The rats (*n* = 6 per group) were sacrificed 7 days post treatment. Mean CFUs with standard deviations are shown. Original CFUs for individual animals are shown in Tables S1A–D. NDC was treated as a 0 value for the calculation of means and standard deviations.

### 3.2 Vancomycin Increased Radiolabeled Antibody Accumulation in the Joint Capsule

**Figure 2:**
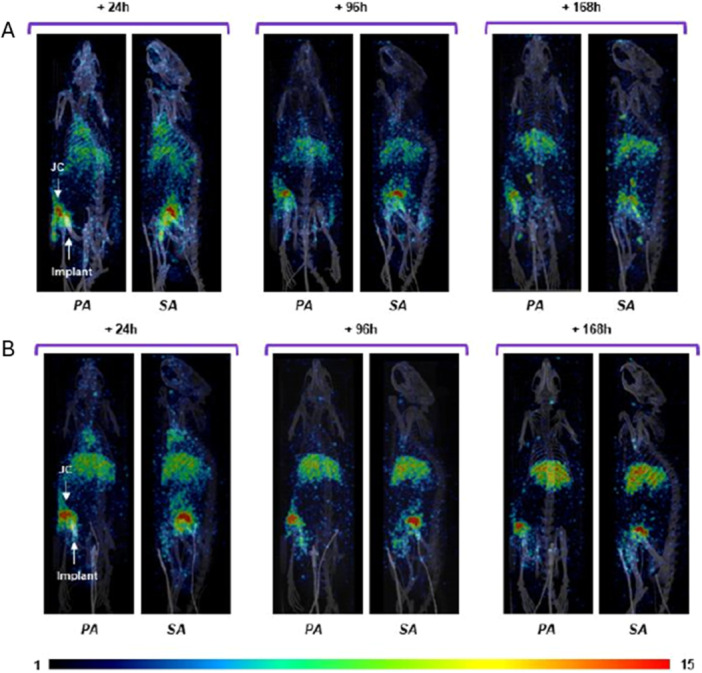
Sequential SPECT/CT imaging of Wistar Han rats with a biofilm‐infected intrafemoral implant injected with ^177^Lu‐4497‐antibody (A) or ^177^Lu‐4497‐antibody+vancomycin (B). The rats were imaged at 24, 96, and 168 h post administration of ^177^Lu‐4497 antibody. Posteroanterior (PA) and Sagittal (SA) views are shown. White arrows point to joint capsule (JC) and implant.

SPECT/CT imaging of ^177^Lu‐4497 in rats with infected implants showed targeting of the infected implant, with ^177^Lu‐4497 uptake in the joint capsule on 24, 96, and 168 h post‐injection for both RIT alone and RIT+vancomycin groups (Figure 2). Sagittal (SA) images revealed predominant radioimmunoconjugate uptake in the joint capsule and synovial space, with delayed penetration into the intramedullary canal. In RIT+vancomycin group increased uptake of ^177^Lu‐4497 in the joint capsule was observed throughout the 7‐day treatment period (Figure 2B).

**Figure 3:**
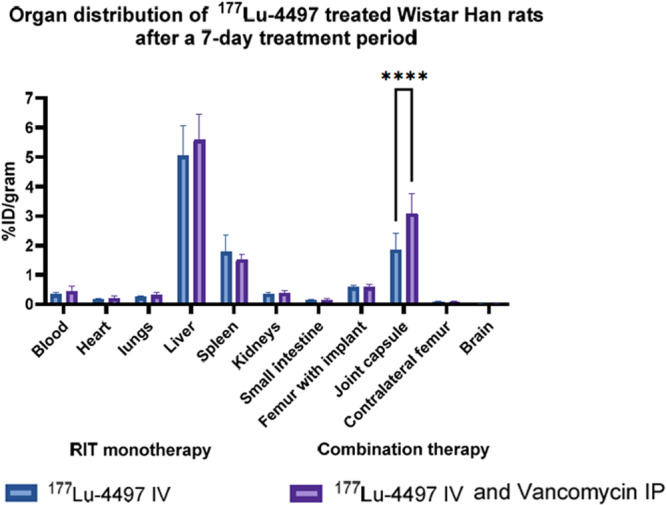
*Ex vivo* biodistribution of ^177^Lu‐4497‐antibody in Wistar Han rats with a biofilm‐infected intrafemoral implant 7 day post treatment. Results are expressed as percentage of the injected dose per gram of tissue (%ID/g). Error bars show standard deviation (SD) of the mean, ****p < 0.0001.

The biodistribution performed at the experiment termination revealed significantly higher accumulation of radioimmunoconjugate in the joint capsule in RIT + vancomycin group compared to RIT alone group with %ID/gram of 3.08 ± 0.68 and 1.85 ± 0.559, respectively (*p* < 0.0001) (Figure 3). The uptake of ^177^Lu‐4497 in the femur with implant and in the contra‐lateral femur was equal for both groups. The liver uptake of ^177^Lu‐4497 was also similar between RIT + vancomycin group and RIT alone groups with %ID/gram of 5.6 ± 0.9 and 5.05 ± 1.0, respectively (Figure 3). Raw %ID/gram can be found in Table S2.

### 3.3 Short‐Term Toxicity Assessment and Gross Pathology of the Joint Capsule

Creatinine, ALT, and albumin levels remained stable, as expected, in both the RIT alone and RIT+vancomycin groups at the experiment termination 7 days post RIT. A slight increase in AST was observed in the control, vancomycin, and RIT+vancomycin groups. Blood urea levels were elevated across all treatment groups (Table 2). Gross pathology of joint capsules revealed possible anti‐inflammatoy effects of RIT as well as vancomycin and RIT + vancomycin (Figure S1). In the control group, the infected joint capsule exhibited pronounced swelling, synovial thikening, hyperemia and accumulation of fluid and pus. In contrast, all treated groups exhibited markedly reduced inflammation signs: reduced hyperemia, diminished swelling, no redness and no fluid accumulation.

**Table 2:** Blood chemistry results after 1 week of follow‐up.

	Control* (*n* = 5)	^177^Lu‐4497 IV (*n* = 6)	Vancomycin IP (*n* = 6)	^177^Lu‐4497 and Vancomycin IP (*n* = 6)	Normal values
Urea	6.9 ± 0.8	6.5 ± 0.7	6.7 ± 0.9	6.9 ± 0.8	2.1−4.10 mmol/L
	(6.1–8.0)	(5.2–7.4)	(5.2–7.9)	(6.0–8.2)	
Creatinine	25.4 ± 3.7	23.4 ± 1.7	22.5 ± 1.9	24.3 ± 2.0	17.7−44.1 μmol/L
	(20–30)	(22–26)**	(20–25)	(22–27)	
ALT	42.6 ± 14.0	37.8 ± 3.5	38.8 ± 17.8	43.2 ± 7.6	18−45 U/L
	(23–62)	(32–41)	(23–73)	(34–56)	
AST	153.8 ± 74.6	109.5 ± 42.0	155.2 ± 111.5	163.8 ± 51.5	74−142 U/L
	(91–281)	(67–173)	(85–382)	(83–225)	
Albumin	3.4 ± 0.13	3.2 ± 0.2	3.1 ± 0.4	3.8 ± 0.2	3.4−4.8 g/dL
	(3.3–3.6)	(2.9–3.4)	(2.6–3.7)	(3.5–4.2)	

*Notes:* Short‐term toxicology assessment was performed with biochemical blood plasma analyses. Values are presented as mean ± SD (range). As expected, due postoperative stress and/or dehydration increased Urea is observed across all treatment groups. No abnormal Creatinine and Albumin was observed for all treatment groups. RIT group showed no liver and kidney function damage. *One blood plasma sample was excluded due to measurement failure (*n* = 5 for this analysis). **Measured in 5 rats due to one failed assay.

## 4 Discussion

This study presents in *vivo* evidence of an antimicrobial effect of RIT on the most prevalent pathogen for implant infections [21] with impressive effect on the planktonic cells in tissue capsule and only some or no effects on the implant and femur, respectively. RIT has been shown to be effective in eradicating pathogens and fungi in vivo and in vitro experiments [16, 22, 23, 24, 25, 26]. As the goal of this study was to compare the bactericidal effect of RIT to that of a course of an antibiotic – we have used CFU quantification which is a gold standard for measuring viable bacteria and assessing clearance in both clinical diagnostics and research [27].

In this study, we observed that single RIT administration achieved a 2.7‐log (99.78%) reduction of bacterial burden in the infected joint capsule, produced no effect on the infected femur and resulted in 72.5% reduction of bacterial burden on the infected implant when compared to untreated control group. Significant bactericidal effect of RIT with ^177^Lu‐4497 antibody on planktonic cells in the joint capsule which migrated there from the biofilm‐infected implant is consistent with our previous observations of ^177^Lu‐4497 being effective against planktonic cells in vitro [26]. We did observe some additive effect of RIT and vancomycin on the implant bacterial burden though it did not reach statistical significance (Table 1) which could be explained by the increased uptake of the radiolabeled antibody in the joint capsule in comparison with RIT alone group causing higher radiation dose to the implant.

The absense of RIT effect on bacterial burden in femur and partial effect on the implant are in contrast with our prior in vitro results when ^177^Lu‐4497 was effective against biofilms. ^26^ However, these observations could be explained by the limitations of the model when the majority of radioconjugate concentrates in the infected joint capsule (Figure 2) which prevents it reaching the intramedullary cavity. In this regard, the epiphyseal and intramedullary regions possess distinct and specialized vascular networks, both of which are likely compromised following intramedullary implant surgery [28] as demonstrated in Wistar Han rats [29, 30]. Although blood flow restoration occurs over time, the inflammatory response following bone injury is characterized by exudate formation, hematoma development, and early angiogenesis [31]. It is likely that, within the first 10 days following surgery, incomplete revascularization and heightened inflammation restrict the penetration of large molecules, such as antibodies, into the intramedullary space [32]. Importantly, that in spite of those challenges a single RIT administration was quite similar in its effectiveness to a 7 day course of vancomycin against planktonic bacterial cells in the joint capsule and bacterial biofilm on the implant.

RIT with ^177^Lu‐4497 did not cause any acute systemic toxicity in treated rats. The elevated urea levels observed across all treatment groups were likely attributable to dehydration rather than renal injury, as creatinine values remained within normal ranges. Gross pathology revealed decrease in inflammation for RIT treated rats similar to the vancomycin and RIT+vancomycin groups. Vancomycin likely reduced the excessive bacteria‐induced inflammation, contributing to vascular stabilization and decreased edema [33, 34], while beta radiation produced by ^177^Lu is known to possess anti‐inflammatory properties, which may help alleviate both postoperative and bacteria‐induced inflammation [35, 36]. Future experiments will incorporate histopathological and immunohistochemical analyses for the markers of inflammation and immune cell invasion to investigate the local immune response to beta‐radiation.

To facilitate the use of RIT in patients with orthopedic implants, improved strategies are required to optimize antibody delivery into the intramedullary space. Direct intramedullary injection or co‐administration of immunomodulatory agents, such as dexamethasone, could help minimize local inflammation and thereby enhance tissue accessibility for therapeutic antibodies. In oncology, increased antibody accumulation in tumors could be achieved by enhancing vascular permeability and blood flow using agents that promote vasculature [37], as well as by co‐administraition of unlabeled antibody [38].

The primary limitation of this study is that rodents may not fully replicate the human condition, particularly with respect to complex inflammatory responses [39] and vascularization. The blood vessels formation in our model is still in acute stage while in patients PJIs take much longer to develop and as a consequence the vascularization of the bone and implant has evolved. However, the biofilms in our model could be considered chronic since they were 6 days old at treatment and 13 days old at experiment termination. Published evidence suggests that rat models can serve as reliable predictors of antibody pharmaco‐kinetics, bioavailability, biodistribution, and clearance [40, 41].

## 5 Conclusions

Effective antibiotic strategies are urgently needed to improve treatment outcomes for persistent biofilm‐associated implant infections. RIT could be a promising and innovative method to eradicate persistent infections, as supported with these findings. Intramedullary implants pose a significant challenge for effective radioimmunoconjugate delivery due to their location within the bone marrow cavity and limited vascular access. However, if these clinical challenges are successfully addressed, RIT could provide a novel and effective strategy for treating biofilm‐associated implant infections.

## Author Contributions

F. Ruben H.A. Nurmohamed, conceptualization, formal analysis, investigation, methodology, writing – original draft, Kevin J.H. Allen, investigation, methodology, software, visualization, writing – review and editing, Mackenzie E. Malo, investigation, supervision, writing – review and editing, Connor Frank, investigation, writing – review and editing, Colleen Nesbitt, investigation, writing – review and editing, J. Fred F. Hooning van Duyvenbode, methodology, writing – review and editing, Michelle Buijs, methodology, writing – review and editing, Berend van der Wildt, methodology, writing – review and editing, Alex J. Poot, methodology, writing – review and editing, Marnix G. E. H. Lam, methodology, writing – review and editing, Jos A. G. van Strijp, methodology, resources, writing – review and editing, Johannes M. van der Merwe, investigation, writing – review and editing, H. Charles Vogely, methodology, writing – review and editing, Harrie Weinans, conceptualization, formal analysis, funding acquisition, methodology, project administration, resources, supervision, writing – review and editing, Bart C.H. van der Wal, conceptualization, funding acquisition, methodology, project administration, resources, supervision, writing – review and editing, Ekaterina Dadachova, conceptualization, funding acquisition, methodology, project administration, resources, supervision, writing – original draft. All authors have read and approved the final submitted manuscript.

## Supporting information

Supporting File

## Data Availability

The data that support the findings of this study are available from the corresponding author upon reasonable request.
