# Supplementary material for: The Effects of Radioimmunotherapy and Antibiotics on Biofilm‐Associated Implant Infections in a Preclinical Rat Model
Source: J Orthop Res. 2026 May 7;44:e70216. doi: 10.1002/jor.70216 (PMC13150401; doi:10.1002/jor.70216)
Supplement: Supplementary file 1 — Supporting File [file JOR-44-0-s001.docx]

**Supplementary Data**

**Supplementary Table S1. Individual doses and CFU counts per treatment group.**

**Table S1, A. Individual dose and CFU of the Control group.**

|  | ***Dose (IV)*** | ***Bacterial count after 7 days*** | | |
| --- | --- | --- | --- | --- |
|  |  | ***Joint capsule*** | ***Femur*** | ***Implant*** |
| Animal #1 | 0.2 ml NaCl | 1.2 x 10^8^ | 5.9 x 10^6^ | 3.8 x 10^4^ |
| Animal #2 |  | 6.1 x 10^7^ | 6.5 x 10^6^ | 4.2 x 10^4^ |
| Animal #3 |  | 3.7 x 10^6^ | 1.0 x 10^6^ | 1.4 x 10^5^ |
| Animal #4 |  | 5.0 x 10^7^ | 2.6 x 10^6^ | 4.3 x 10^6^ |
| Animal #5 |  | 7.8 x 10^6^ | *Lost sample* | 1.2 x 10^5^ |
| Animal #6 |  | 1.6 x 10^7^ | 4.1 x 10^6^ | 1.91 x 10^7^ |
| *Seven days after treatment with a single injection of NaCl bacterial colonization of the joint capsule, surrounding femoral bone, and implant was assessed. Tissue samples were homogenized and subjected to tenfold serial dilutions, which were plated in duplicate on Columbia Blood Agar. After overnight incubation at 37°C, colony-forming units (CFU) were counted. Mean values are reported. NDC: No Detectable Colonies.* | | | | |

**Table S1, B. Individual dose and CFU of the** **RIT monotherapy group.**

|  | ***RIT Dose (IV)*** | ***Bacterial count after 7 days*** | | |
| --- | --- | --- | --- | --- |
|  |  | ***Joint capsule*** | ***Femur*** | ***Implant*** |
| Animal #1 | 118.8 MBq/kg | *NDC* | 5.4 x 10^6^ | 1.3 x 10^5^ |
| Animal #2 | 116.3 MBq/kg | *NDC* | 1.2 x 10^7^ | 4.4 x 10^4^ |
| Animal #3 | 116.1 MBq/kg | *NDC* | 1.6 x 10^7^ | 1.3 x 10^5^ |
| Animal #4 | 114.7 MBq/kg | *NDC* | 8.0 x 10^6^ | 7.6 x 10^4^ |
| Animal #5 | 116.1 MBq/kg | 5.6 x 10^5^ | 5.6 x 10^7^ | 3.3 x 10^5^ |
| Animal #6 | 117.5 MBq/kg | 1.0 x 10^3^ | 2.0 x 10^7^ | 5.7 x 10^6^ |
| *Seven days after intravenous administration of RIT (mean dose of 116.6 MBq/kg), bacterial colonization of the joint capsule, surrounding femoral bone, and implant was assessed. Tissue samples were homogenized and subjected to tenfold serial dilutions, which were plated in duplicate on Columbia Blood Agar. After overnight incubation at 37°C, colony-forming units (CFU) were counted. Mean values are reported. NDC: No Detectable Colonies.* | | | | |

**Table S1, C. Individual dose and CFU of the Vancomycin monotherapy group.**

|  | ***Vancomycin Dose (IP)*** | ***Bacterial count after 7 days*** | | |
| --- | --- | --- | --- | --- |
|  |  | ***Joint capsule*** | ***Femur*** | ***Implant*** |
| Animal #1 | 88 mg/kg | *NDC* | *NDC* | 4.5 x 10^4^ |
| Animal #2 |  | *NDC* | 1.0 x 10^6^ | 1.4 x 10^4^ |
| Animal #3 |  | *NDC* | *NDC* | 2.3 x 10^6^ |
| Animal #4 |  | *NDC* | *NDC* | 2.5 x 10^4^ |
| Animal #5 |  | *NDC* | *NDC* | 2.3 x 10^4^ |
| Animal #6 |  | *NDC* | *NDC* | 6.9 x 10^4^ |
| *Seven days after treatment with vancomycin (intraperitoneal injection, 88 mg/kg), bacterial colonization of the joint capsule, surrounding femoral bone, and implant was assessed. Tissue samples were homogenized and subjected to tenfold serial dilutions, which were plated in duplicate on Columbia Blood Agar. After overnight incubation at 37°C, colony-forming units (CFU) were counted. Mean values are reported. NDC: No Detectable Colonies.* | | | | |

**Table S1, D. Individual dose and CFU of the Combination Therapy group.**

|  | ***RIT Dose (IV)*** | ***Vancomycin Dose (IP)*** | ***Bacterial count after 7 days*** | | |
| --- | --- | --- | --- | --- | --- |
|  |  |  | ***Joint capsule*** | ***Femur*** | ***Implant*** |
| Animal #1 | 118.5 MBq/kg | 88 mg/kg | *NDC* | 6.4 x 10^6^ | 2.7 x 10^4^ |
| Animal #2 | 118.0 MBq/kg |  | *NDC* | *NDC* | 1.4 x 10^4^ |
| Animal #3 | 114.3 MBq/kg |  | *NDC* | 9.9 x 10^5^ | 6.2 x 10^4^ |
| Animal #4 | 114.2 MBq/kg |  | *NDC* | *NDC* | 4.1 x 10^4^ |
| Animal #5 | 118.5 MBq/kg |  | *NDC* | 3.5 x 10^5^ | 4.5 x 10^4^ |
| Animal #6 | 117.1 MBq/kg |  | *NDC* | *NDC* | 2.8 x 10^4^ |
| *Seven days after intravenous administration of low-dose RIT (mean dose of 116.8 MBq/kg), and treatment with Vancomycin (intraperitoneal injection, 88 mg/kg), bacterial colonization of the joint capsule, surrounding femoral bone, and implant was assessed. Tissue samples were homogenized and subjected to tenfold serial dilutions, which were plated in duplicate on Columbia Blood Agar. After overnight incubation at 37°C, colony-forming units (CFU) were counted. Mean values are reported. NDC: No Detectable Colonies.* | | | | | |

**Supplementary Table S2. Raw Biodistribution Data (%ID/gram) of all ^177^Lu-4497 treated rats.**

|  | | ***^177^Lu-4497 IV***  ***N=6*** | | | ***^177^Lu-4497 IV and Vancomycin IP N=6*** | |
| --- | --- | --- | --- | --- | --- | --- |
|  | ***Mean*** | | ***SD*** | ***Mean*** | | ***SD*** |
| ***Blood*** | 0.329831 | | 0.095 | 0.446588 | | 0.164 |
| ***Heart*** | 0.16768 | | 0.037 | 0.213119 | | 0.086 |
| ***Lungs*** | 0.249176 | | 0.049 | 0.318224 | | 0.097 |
| ***Liver*** | 5.050249 | | 1.000 | 5.572858 | | 0.899 |
| ***Spleen*** | 1.783548 | | 0.559 | 1.517408 | | 0.185 |
| ***Kidneys*** | 0.356654 | | 0.039 | 0.385341 | | 0.095 |
| ***Small Intestine*** | 0.136609 | | 0.035 | 0.135327 | | 0.049 |
| ***Femur with implant*** | 0.588271 | | 0.052 | 0.582974 | | 0.108 |
| ***Joint capsule*** | 1.84978 | | 0.559 | 3.084811 | | 0.679 |
| ***Contralateral femur*** | 0.086889 | | 0.012 | 0.092425 | | 0.017 |
| ***Brain*** | 0.012322 | | 0.004 | 0.019421 | | 0.010 |


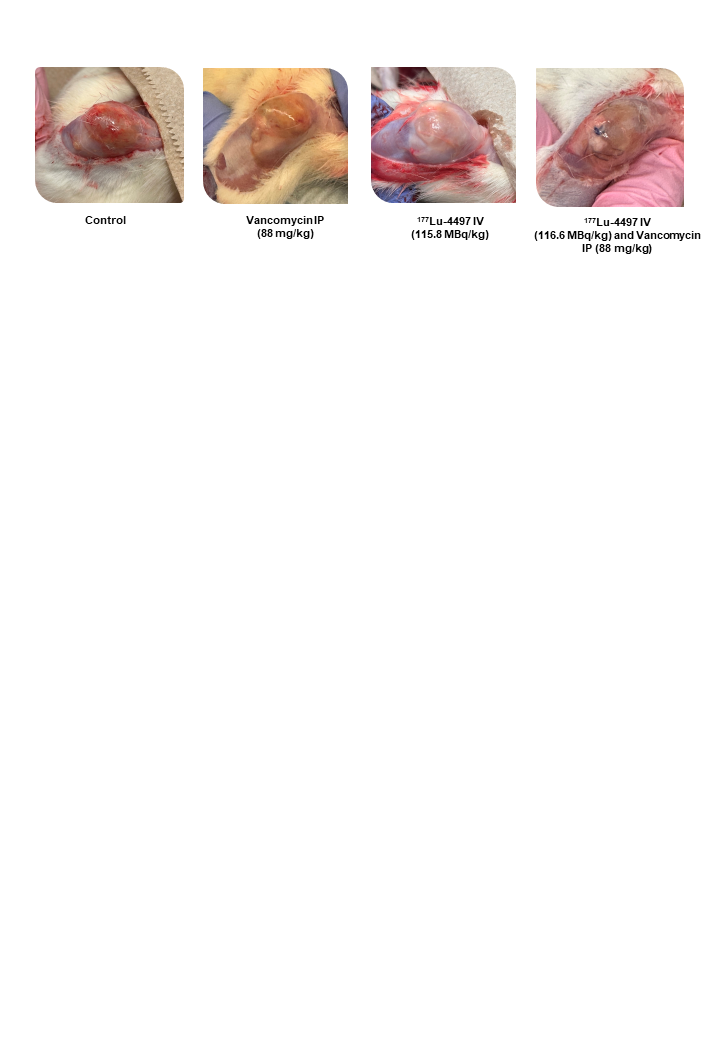


**Supplementary Fig. S1. Visual comparison of joint capsule inflammation across treatment groups after a 7-day treatment period.** Images were captured post-mortem. In the control group, joint tissue appeared swollen, red, and filled with pus and fluid, indicating not only a substantial bacterial infection but also a pronounced bacteria-associated inflammatory response. In the vancomycin group (no detectable bacterial growth after 7 days) and RIT alone group, inflammation was visibly reduced, with diminished redness and swelling. Notably, in RIT+vancomycin group (no detectable bacterial growth after 7 days), joint tissue appeared normal, showing no signs of swelling, redness, or fluid accumulation. These findings suggest a beneficial anti-inflammatory effect of β-radiation when combined with antibiotic treatment.
